# Progress in Surgery

**Published:** 1895-02-09

**Authors:** 


### Progress in Surgery.

### GENERAL SURGERY.

(*Continued from page* 314.)

**Fractures and Dislocations.**—In the treatment of compound fractures, surgeons now hold that no matter how extensively bone is injured, in the absence of other complications this constitutes no ground for amputation. The bone should be treated as if there was no injury to the soft parts; the soft parts as if there was no injury to the bone. Crile[21] considers that (unless under continuous observation) plaster of Paris bandages should not be applied until the acute symptoms have subsided and the condition of the wound has declared itself. The fragment should be thoroughly reduced, and if necessary held in place by strong silk sutures (either periosteal or osseous). In cases of infection ample dependent drainage should be provided, and the wound and limb thoroughly cleansed. In a recent discussion at the Chirurgical Society of Paris, on the treatment of fractures by osseous suture, Dr. Nélaton[22] did not urge this method as a routine treatment of grave fractures. He said that there had been cases in which patients were afflicted with a crooked leg on account of vicious consolidation, resulting in impaired function which might have been avoided by resorting to the method employed by Roux de Brignoles. He thinks we are both justified, and in duty bound, to make use of some form of osseous suture in certain cases of irreducible and intractable fracture. In five cases J. B. Hamilton[23] has used the bone ring introduced by Senn. In every case there was suppuration, and he has abandoned it until such time as we shall be enabled to sterilise the ring. It is yet so far from perfection in this respect as to make it almost certain that the wound will become infected. In compound fractures of the shaft of the femur Llewellyn Eliot[24] advises suturing or pegging and treatment of the wound by the open method. With regard to simple fractures he calls attention to the fact that Benjamin Gooch, of London, in 1780, was the first to employ adhesive plaster as a means of extension. This combined with the proper application of splints will generally be satisfactory, although shortening occurs in very many cases. On the other hand in a paper on the treatment of fractures, Dr. Maclean[25] quotes a paper by James Syme in 1861, in which he entirely discredited the practice of trying to treat fractures of the femur by extension and counter-extension, first on the ground that it is impossible to produce the end sought, and, second, that it is quite unnecessary. Eliot follows Symes' example by treating these as well as fractures of other bones without extension. Cunnane[26] reports a case of fracture of the pelvis and femur with laceration of the bladder terminating in recovery. Fracture of the pelvis is almost unknown in children, but in this instance it occurred in a girl aged five years. An interesting specimen of impacted subtrochanteric fracture of the femur is figured by Gouley.[27] It had been diagnosed during life by the character of the deformity, swelling below the hip, rotation inwards and adduction of the limb, degree of shortening ($1\frac{1}{2}$ in.), and the absence of crepitus. These led the house surgeon to believe at first that it was a case of post-iliac dislocation of the femur. Disimpaction of the fragments was effected during life, and believed to be justified on account of the extent of the deformity. The patient, aged 46, died on the ninth day from the effects of chronic alcoholism. Irvine[28] gives a note on the importance of the nutrient artery and vein for the shaft of the tibia in regard to its vitality, as illustrated by the effects of an apparently trivial injury to that bone in a man of 66 years of age. Two and a half inches of the tibial shaft became necrosed, but ultimate recovery took place. A fatal case of fat embolism after simple fracture of the tibia and fibula, through interference with the pulmonary circulation, is recorded by Hirsch,[29] though such complication is denied by Scriba and others. Fracture of the head of the fibula in a case reported by Horton[30] is believed to have been caused by muscular action, viz., by the violent contraction of the biceps. It occurred in a man aged 64. At the twenty-third Congress of German Surgeons Bardeleben[31] stated that he had tried ambulatory dressings or splints in 116 fractures of the thigh or leg, including complicated as well as simple fractures with very satisfactory results. There was no fear of tumefaction in the limb; practically it never occurred, especially when care was taken to apply the apparatus before it had time to make its appearance. The ambulant treatment is of special importance in the case of the aged and among alcoholics. Bones unite more readily than if kept at rest. A gelatin plaster of Paris was used with iron bands to give the required firmness. The cases began to walk the day after the leg was put up. The treatment was applicable even in compound fractures, provided the wound could be rendered completely aseptic at the outset. In an interesting paper, entitled "Sprains and Disabled Joints," Bradford[32] says that fixation by apparatus can be necessary only to stop the pain for a short time after a severe sprain. In the early stages motion, short of causing pain, is harmless; in the later stages motion and use should be allowed up to the point of pain; but in some instances, in highly sensitive persons, guarded and protracted motion, even beyond the point of pain, is indicated. Compression may be useful for a short time during the period of effusion, but after that it becomes injurious. The application of heat and cold and massage improve the circulation in the parts about the joint. A. C. Wiener[33] also advocates the Lucas Championnière treatment of fractures and sprains of the ankle by massage. Although that method is receiving more attention in America since the publication of Championnière's Thesis in 1886, it does not appear to be so generally recognised as in France. The irreducibility of subastragular dislocation, according to a specimen presented by Dr. Quéner[34] to the Chirurgical Society of Paris, is explained by the head of the astragalus passing through the anterior ligament of the tarsus. The dislocation from his experiments is produced by strong flexion of the foot or the leg. Therefore, for reduction, the foot must not be extended as Malgaigne contends, but on the contrary, forcibly flexed, and direct pressure applied to the astragalus.

If this does not succeed, the anterior ligament should be divided. Cases of extirpation of the astragalus should be greatly reduced in number. McBurney and Dowd[35] recommend a new method of reduction of those very rare cases of dislocation of the humerus complicated by fracture at or near the surgical neck. An incision is carried through the deltoid muscle to the outer surface of the upper fragment; with a drill a hole is then made through the bone and a traction hook inserted. By the means of the latter the humeral head may be easily reduced, though considerable force is required. McBurney records a most satisfactory case of a man, aged forty-eight, in whom, after reduction of the dislocation, the apposition of the fragments was so perfect that no osseous suture was required. Primary union occurred, and on the twenty-fourth day the plaster of Paris splint was finally removed. The fragments having united passive motion was commenced, and the ultimate functional results were perfect. A very rare specimen of fracture of the coracoid process, involving the articular surface of the glenoid cavity of the scapula, is figured by S. E. Jones[36], taken from a woman aged fifty-two. There were the usual signs of fracture of the coracoid process during life, and death ensued from head injuries five days after the accident. Some considerations about injuries involving the elbow joint formed the subject of a recent interesting lecture by Sir William MacCormac[37]. After alluding to the numerous varieties of injury which often render the diagnosis difficult, and the frequency of impairment of joint function afterwards, MacCormac emphasises the fact that in many of these cases the change in the shape of the articular surface as the result of fracture in itself greatly militates against a satisfactory result as regards function. In many cases, whatever the treatment may be, a great deal of alteration in the shape of the joint may ensue. The uniform practice at St. Thomas's Hospital is to treat these joint fractures in the flexed position. A plaster of Paris splint is used to fix the limb until union has taken place, or until a certain amount of repair has occurred, then passive movements are resorted to. Cook[38] and Rotter[39] advocate the same lines of treatment in fractures into joints, not only because of the relief it gives to pain, but because by this means the lacerated parts are held in their proper position until repaired. Morton[40] has practised a new method for reduction of fracture of the lower end of the radius by means of superextension by the surgeon's hands combined with powerful traction upon the wrist. The patient's hand is then fully flexed and the displaced lower fragment powerfully pressed downwards into position by the surgeon's thumb. Dr. Ybarra[41] contributes a valuable paper on the wounds of the Mannlicher magazine rifle as exemplified in the recent civil war in Chili, with general remarks on all the modern military small-bore rifles. The wound made in the shaft of long bones with the bullet of the Mannlicher rifle at full speed was very much as if made with a boring machine, so clean cut, straight, and regular throughout was the track left. Gunshot wounds of long bones cause, as a rule, fractures which are rarely simple. When the Mannlicher bullet happened to strike an epiphysis, it usually caused fissures; however, one instance was observed in which the projectile passed clean through, leaving a perfectly cylindrical track, without any splintering of the bone or the formation of fissure, the wound healing by first intention at the end of ten days. In a few rare cases some explosive effects were noticed.

[21] Therapeutic Gazette, July 16th, 1894. [22] Medical Week, June 22nd, 1894, p. 295, and June 29th, p. 308. [23] N. Y. Med. Jour., Aug. 11th, 1894, p. 192, and Journal of Amer. Med. Asso., July 21st. [24] Therapeutic Gazette, Sept. 15th, 1894, p. 595. [25] Chicago Clinical Review, Sept., 1894, p. 681. [26] N. Y. Med. Record, July 21st, 1894 p. 72. [27] N. Y. Med. Jour., Aug. 11th, 1894, p. 184. [28] Edinburgh Med. Jour., Oct., 1894, p. 326. [29] Brit. Med. Jour., July 7th, 1894, p. 13. [30] N. Y. Med. Record, Sept. 15th, 1894. [31] N. Y. Med. Jour., July 7th, 1894, and Gazette Hebdomadanet de Méd. N. de Chirurg., June 16th. [32] Ibid., Sept. 1st., 1894, p. 257. [33] N. Y. Med. Record, Sept. 22nd, 1894, p. 371. [34] Med. Week, May 25th, 1894, p. 246. [35] Annals of Surgery, April, 1894, p. 399. [36] The Hospital, Aug. 25th, 1894, p. 428. [37] The Clinical Journal, Oct. 10th, 1894, p. 382. [38] Amer. Med. and Surg. Bulletin, Oct. 1st, 1894. [39] Therapeutic Gazette, Aug. 15th, 1894, p. 563. [40] N. Y. Med. Jour., July 7th, 1894, p. 26. [41] International Med. Mag., July, 1894, p. 415.

